# Supplementary material for: Development of a Functional Cookie Formulated with Chaya (Cnidoscolus aconitifolius (Mill.) I.M. Johnst) and Amaranth (Amaranthus cruentus)
Source: Molecules. 2022 Oct 31;27(21):7397. doi: 10.3390/molecules27217397 (PMC9658788; doi:10.3390/molecules27217397)
Supplement: Supplementary file 1 [file molecules-27-07397-s001.zip › molecules-1991822-supplementary.pdf]

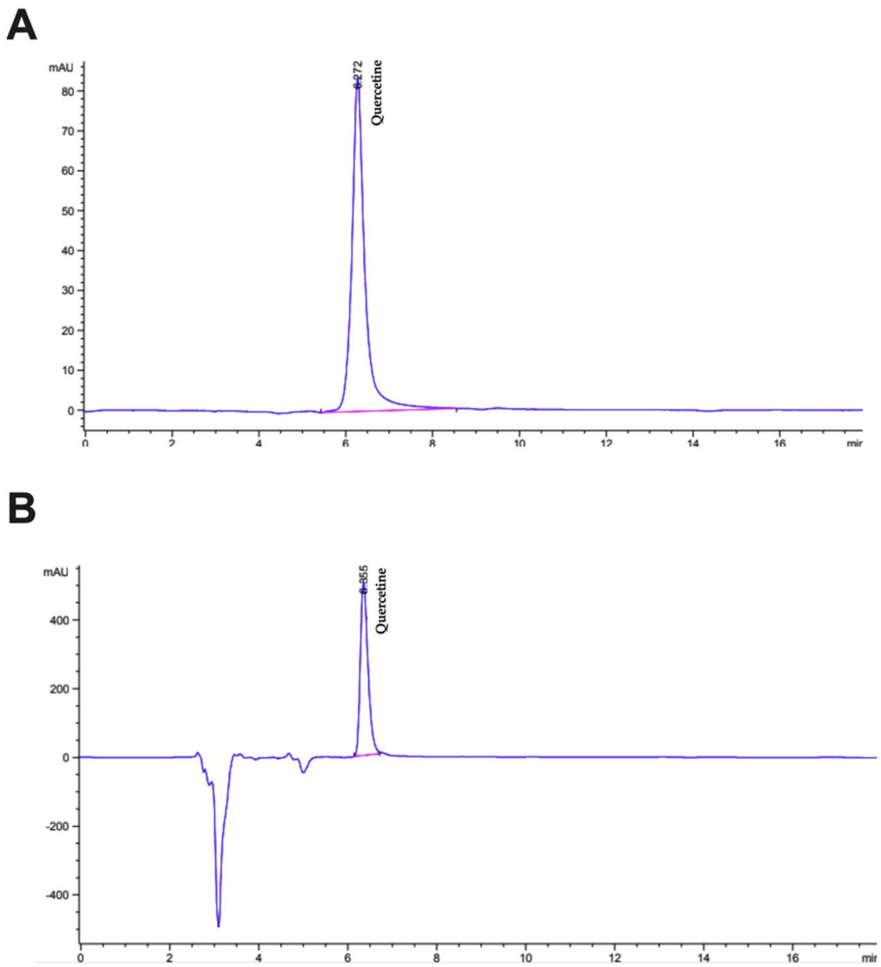


**Figure S1:** Profile by HPLC of (**A**) quercetin standard and (**B**) chaya flour.

**Table S1.** Nutritional Facts of formulated cookies.

| | Formulation | | | | | | | |
|---|---|---|---|---|---|---|---|---|
| | **Ch5:A20** | | **Ch10:A15** | | **Ch15:A10** | | **Ch20:A5** | |
| **Calories*** | | 147 | | 145 | | 145 | | 146 |
| | **g/30g** | **%Daily value** | **g/30g** | **%Daily value** | **g/30g** | **%Daily value** | **g/30g** | **%Daily value** |
| **Total Fat** | 5 | 7 | 5 | 7 | 5 | 7 | 5 | 7 |
| **Saturated Fat** | 0.05 | 0.25 | 0.05 | 0.23 | 0.05 | 0.25 | 0.05 | 0.24 |
| **Trans Fat** | 0 | | 0 | | 0 | | 0 | |
| **Cholesterol** | ♦ND | | ♦ND | | ♦ND | | ♦ND | |
| **Sodium** | 2 | 98 | 1 | 65 | 1 | 45 | 1 | 53 |
| **Total carbohydrates** | 22 | 8 | 21 | 8 | 21 | 8 | 21 | 8 |
| **Dietary Fiber** | 2 | 7 | 2 | 7 | 2 | 6 | 2 | 6 |
| **Total Sugars** | 20 | | 19 | | 20 | | 20 | |
| **Protein** | 3 | 6 | 3 | 6 | 3 | 6 | 3 | 6 |

*Theorical determination of caloric content; ♦ No Determined
